# Putative effects of moringa oil or its nano-emulsion on the growth, physiological responses, blood health, semen quality, and the sperm antioxidant-related genes in ram

**DOI:** 10.1186/s12917-024-04444-7

**Published:** 2025-01-09

**Authors:** Rehab F. S. A. Ismail, Wael A. Khalil, Sara I. Grawish, Karima Gh. M. Mahmoud, Sameh A. Abdelnour, Alaa M. A. Gad

**Affiliations:** 1 Department of Animal Production, Faculty of Agriculture, Mansoura University, Mansoura, 35516 Egypt; 2 Animal Production Research Institute, Agriculture Research Centre, Ministry of Agriculture, Dokki, 12619 Giza Egypt; 3 Department of Animal Reproduction and AI, Veterinary Research Institute, National Research Centre, Dokki, 12622 Egypt; 4 Department of Animal Production, Faculty of Agriculture, Zagazig University, Zagazig, 44511 Egypt

**Keywords:** Ram, Semen attributes, Blood health, Moringa oil and its nano-form

## Abstract

Phytochemicals have been effectively used to enhance the growth and productivity of farm animals, while the potential roles of essential oils and their nano-emulsions are limited. This plan was proposed to investigate the impacts of orally administered moringa oil (MO) or its nano-emulsion (NMO) on the growth, physiological response, blood health, semen attributes, and sperm antioxidant-related genes in rams. A total of 15 growing Rahmani rams were enrolled in this study and allotted into three groups. The 1^st^ control group received a basal diet only and treated orally one mL of distilled water, while the 2^nd^, and 3^rd^ groups received a basal diet and were orally treated with 1 mL of NMO or 2 mL of MO /head/day for 4 months, respectively. Growth, physiological response, blood health, semen quality, and antioxidant genes in sperm were assessed. The MO and NMO treatments had no significant effect on growth indices (final body weight and weight gain ) and physiological response (rectal temperature, pulse, and respiration rates) (*P* > 0.05). The NMO group had the lowest levels of MCV (mean corpuscular volume) (*P* < 0.05), while all treated groups produced higher levels of mean corpuscular hemoglobin (MCH) and mean corpuscular hemoglobin concentration (MCHC) compared to those in the control group (*P* < 0.05). Aspartate transferase (AST) and total cholesterol were significantly reduced in the MO and NMO groups, while total protein and glucose levels were significantly improved in NMO group (*P* < 0.05). Serum and seminal interstitial-cell-stimulating hormone (ICSH) levels were significantly improved (*P* < 0.0001) in the NMO group. Testosterone in serum and seminal plasma was significantly improved (*P* < 0.0001) in the MO group. Total antioxidant capacity (TAC) levels showed a tendency to increase in both the MO and NMO groups, but this increase was not significant compared to the untreated group (*P* > 0.05). On the other hand, the MO group exhibited lower levels of AST and malondialdehyde (MDA), while the alanine aminotransferase (ALT) levels were the lowest in the NMO group (*P* > 0.05). Mass motility, viability, membrane integrity and sperm concentration were significantly improved in the MO group (*P* < 0.0001) compared to the other groups. The NMO group had worse expressions of *superoxide dismutase 1 (SOD1)* compared to the control and MO groups. MO group significantly upregulated the *catalase* gene compared to the other groups (*P* < 0.001). The expression of *Caspase-3* was highest in the group that received NMO compared to the other groups (*P* < 0.001). This study suggests that MO may serve as a novel therapeutic agent for improving the reproductive health in Rahmani rams.

## Introduction

Globally, the sheep population is approximately 1.266 billion, and has a significant impact on the livestock sector [1]. There are several factors affecting the profits and productivity of sheep farm such as environmental elements, genetic feeding regimes, and reproductive capacity [2–4]. Reproductive performance is a critical variable affecting flock profitability with reproductive health of the growing rams acting an important roles in this aspect [4]. Actually, 50% of the reproductive capacity of a sheep herd is promoted by the ram [4]. Efficient animal reproduction management is essential for optimizing economic and productivity outcomes and ensuring animal longevity [5, 6]. Selecting and acquiring males, as well as efficiently managing their mating behavior during pre- and post-breeding seasons, present challenges for utilizing males in both natural and artificial insemination process [7, 8]. Male infertility is affected by the detrimental impacts of oxidative stress (OS) induced by environmental issues or malnutrition, which impairs sperm function development [9, 10]. The antioxidant defense system can protect body cells against the harmful effects of OS triggered by external factors, thereby preventing male infertility [11]. Additionally, undernutrition has significant impacts on reproductive capacity in sheep [10], and can reduce health and welfare. Therefore, several attempts have been made to improve the health, welfare, and fertility outcomes in rams under extensive farm systems through dietary interventions.

Nowadays, herbal remedies are widely used worldwide as an alternative to pharmaceutical medicines. Feed additives containing herbs rich in active constituents can provide an ecological and natural approach to enhancing the reproductive capacity of farm animals [2, 12]. Moringa oil (MO) is extracted from the seeds of *the Moringa oleifera*, a fast-growing tree found in Africa, the Middle East, and Asia. Recent studies have highlighted the health benefits, excellent oxidative stability, and strong antimicrobial properties of MO [13].

However, the effectiveness of MO in inhibiting bacterial growth over an extended period is limited due to its volatility and sensitivity to light, air, and high temperatures. Therefore, to improve the stability and prolong the shelf life of MO, nanoencapsulation technology was used to encapsulate MO and prevent the loss of active constituents. *Moringa oleifera* has been widely used as feed for ruminants, with significant effects on health, growth, microbiota, milk quality, and feed utilization in small ruminants [14–16]. *Moringa oleifera* is considered a promising option for animal feed, but its potential benefits for improving health, redox status, and semen quality of rams have not been thoroughly investigated. This study aims to explore the effects of *Moringa oleifera* oil or its nano-emulsion on body weight, hormonal profile, antioxidant and oxidative biomarkers in serum and seminal plasma, as well as sperm quality of fresh semen in rams. This research seeks to examine the potential impacts of *Moringa oleifera* seed oil (MO) or its nano-emulsion (NMO) on ram health and reproductive parameters.

## Material and methods

### Preparation of moringa oil nano-emulsion

The moringa oil was obtained from Pure Life Company in Giza, Egypt. The nano-emulsion of moringa oil was prepared using a surfactant mixture (Tween 80) by gradually adding water via a magnetic stirrer at 25 °C. The rate of water addition was kept constant at approximately 1.0 mL/min. The emulsion was then dispersed by sonication for 30 min using an ultrasonic bath (Sonix USA, SS101H230) and further homogenized using an ultrasonic probe (Serial No. 2013020605) attached to a homogenizer (Sonics Vibra-cell™, Model VC 505, USA) under the following conditions: Amplitude: 60%, Timer: 3 min, Pulser: 1 s ON/1 s OFF, to produce nano-emulsions of moringa oil.

### Determination of moringa oil nano-emulsion

**Fig. 1:**
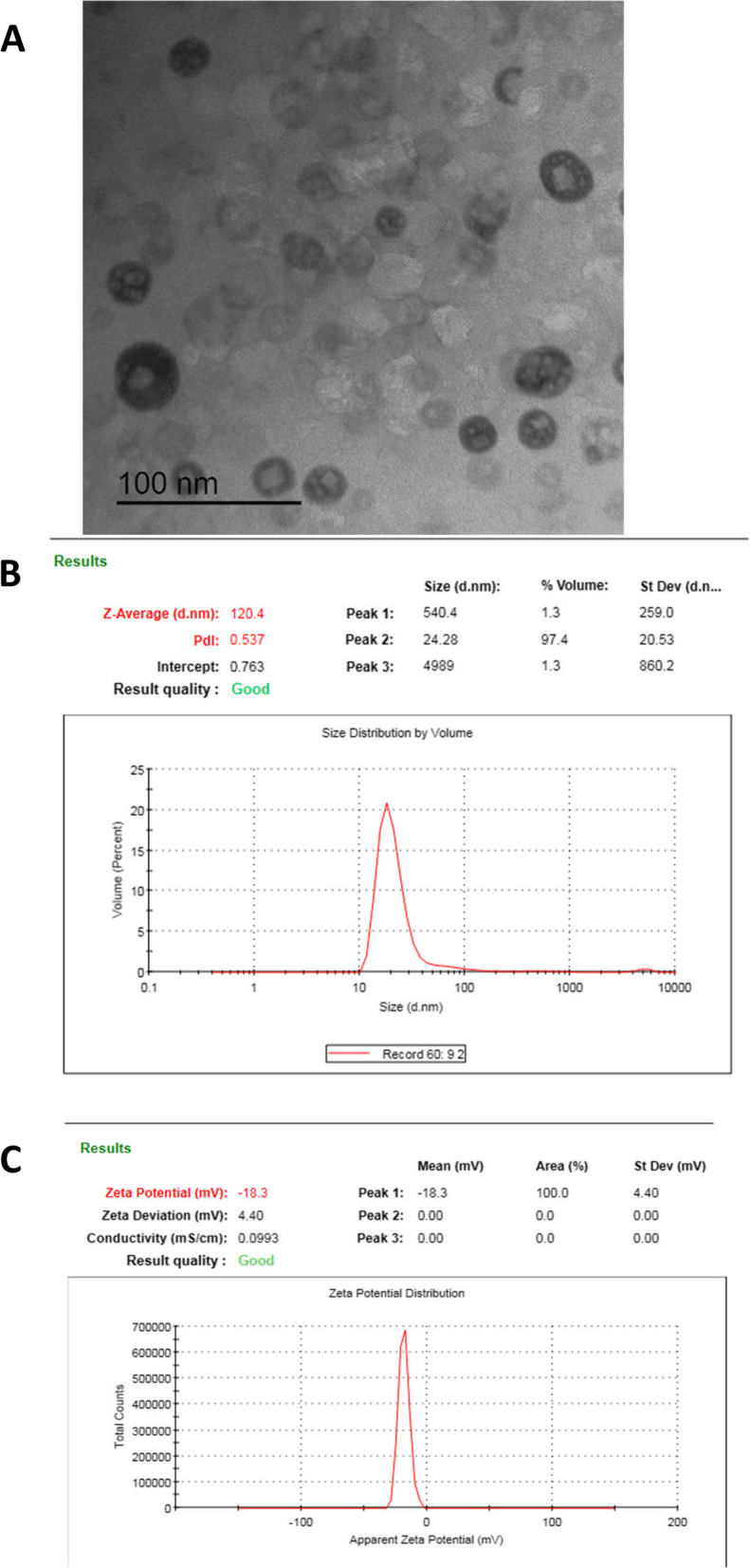
TEM morphology of moringa oil nano-emulsion (NMO) A, particles size B, and zeta potential C

To confirm the synthesis of MO nano-emulsion, the average vesicular size (Z-average) and polydispersity index (PDI) were evaluated using a Zetasizer Nano ZS analyzer (Malvern Instruments, Malvern, UK). The measurements were conducted in triplicates after adequate dilution with distilled water (1:10) of the freshly prepared formula at 25 °C. Additionally, the morphology of the MO nano-emulsion (NMO) was visualized by transmission electron microscopy (TEM). The freshly prepared moringa oil nano-emulsion was imaged using a TEM (JEOL JEM-2100, JEOL Ltd., Tokyo, Japan). After dilution with distilled water and sonication, the carbon-coated copper grid was coated with the moringa oil nano-emulsion dispersion, air-dried at room temperature, and directly inspected using TEM at 160 kV without staining. The image capture, analysis process, and particle sizing were completed using Digital Micrograph and Soft Imaging Viewer software (Gatan Microscopy Suite Software, version 2.11.1404.0). The characterization of the MO nano-emulsion is presented in Fig. 1.

*Ethical approval.*

This research was approved by the Animal Ethics Committee of Zagazig University (Approval No. ZU-IACUC/2/F/25/2023) and in compliance with the ARRIVE guidelines. The study was carried out at the Farm of Department of Animal Production, Faculty of Agriculture, Mansoura University, Egypt.

### Animal’s management and experimental design

Fifteen healthy growing Rahmani males (6 months old and weighing 30.6 ± 0.5 kg) were included in this experiment to investigate the potential effects of MO or its nano form (NMO) on enhancing the reproductive efficiency of rams. The animals (*n* = 15) were randomly divided into three experimental groups, with five animals in each group. The first group served as the control (without supplementation; orally administered with 1mL of distilled water). The second group was the NMO group, which received NMO at a dose of 1 mL/head/day, and the third group was the MO group, where rams received MO at a dose of 2 mL/head/day. All treated animals received the treatment via individual oral administration before morning feeding. The animals were fed ad libitum, with a feed concentrated with a protein ratio of 16% diets provided at a rate of 3% of the animal’s weight, and filler diets provided at a rate of 1% of the animal’s weight (red hay or wheat hay), and alfalfa hay provided at a rate of 1.5% of the animal’s weight, based on NRC recommendations. The feed was offered twice daily at 8:00 am and 5:00 pm, with free access to fresh water. The diet composition is shown in Table 1. The Rahmani lambs were housed in semi-shaded barns with subdivisions. An adaptation period of two weeks was implemented before initiating treatments, followed by a four-month study duration. The experimental animals were treated against internal and external parasites using an anthelmintic drug before starting the trial.

**Table 1:** Composition of the experimental basal diet

Items	Percentage	Items	Percentage
Corn	40	Di calcium phosphate	0.8
Wheat bran	22	Premix fattening	0.3
Soybean meals	10	Sodium bicarbonate	0.2
Gluten	3.5	Molasses	2.65
Glutamine	2.5	Antitoxin	0.1
polygons	10	Antihelminthic	0.05
Sunflower meals	5	Live yeast	0.05
Limestone	2	Immunity booster	0.05
Table salt	0.8		

### Environmental conditions

The relative humidity and ambient temperatures (°C) were evaluated daily throughout the trial using an automatic thermo-hygrometer (Dostmann GmbH, Wertheim, Germany) set up on the farm. The temperature-humidity index (THI) helps in understanding the severity of environmental temperatures on animals. The procedure for analyzing THI, as summarized by Marai et al. [17], is as follows:

THI = (1.8 × Tdb + 32)—[(0.55—0.0055 × RH) × (1.8 × Tdb—26.8)].

Here, Tdb represents the dry bulb temperature in Celsius and RH is the relative humidity in percentage divided by 100, applicable for sheep and goats. The stress levels for ruminants (sheep and goats) based on THI [18], are as follows: comfortable ≤ 72; mild stress 73–78; severe stress ≥ 80. 

### Body weights and weight gain

The live weights of each animal were assessed before feeding in the morning at the starting point of the trial (day 0) and at the end of each month of experimental trial (4 months).

### Thermoregulatory responses

A digital thermocouple (Type K) was inserted 3cm in the rectum of rams to measure the rectal temperature. The respiration rate was also assessed through counting the number of breaths/min in each animal. Pulse rate was assessed using the pulsometer.

### Blood sampling collection

At the end of the experimental period (day 120), blood specimens were gathered from the jugular vein of each ram into sterilized tubes. The samples were divided into subsamples, the first one was kept in EDTA-tube for hematology assessment. The second sub-sample was transferred into centrifugation tubes and left in the room to coagulate. To set the serum, the samples were subjected to centrifugation at 4000 rpm for 10 min at room temperature. The serum samples were collected and stored in 2 mL vials at -20°C until they were analyzed.

### Hematology assessment

The whole blood was used for assessing RBCs (red blood cells, X10^6^ /mm^3^), hemoglobin (Hb, g/dL), Hct (hematocrit, %), mean corpuscular volume (MCV, μm^3^), mean corpuscular hemoglobin (MCH, pg), mean corpuscular hemoglobin concentration (MCHC, g/dL), platelets (PLT, X10^3^ /mm^3^), WBCs (white blood cells, X10^3^/ mm^3^), lymphocytes (%) and neutrophils (%) using Veterinary Auto Hematology Analyzer (Model: BK-3200VET; BIOBASE Company, Shandong, China).

### Serum metabolites

Liver and kidney functions, lipid profiles and oxidative biomarkers were assessed from all animals in each group. The serum levels of aspartate transaminase (AST), alanine transaminase (ALT), glucose, albumin, bilirubin, total proteins, creatine, urea, total cholesterol, total glycerides, total antioxidant capacity (TAC) and malondialdehyde (MDA), were assessed using commercial kits (Biodiagnostic, Giza, Egypt) and a spectrophotometer (Spectro UV–Vis Auto, USA) following the instructions provided based on the company.

### Semen collection

For the semen collection, commencing in the fourth month of the experiment, the rams were trained for semen collection. Examination and analysis of the samples began in the fifth month of the experiment. Semen samples (n = 60; one sample per animal at each time point, 15 × 4) were collected in the early morning using an artificial vagina and adjusted to 42°C with lubrication. Immediately after collection, the ejaculates were transported to the laboratory and placed in a warm water bath at 37°C. The semen samples were then evaluated using standard laboratory procedures. The semen samples were assessed immediately after the collection for volume, mass motility, viability, membrane integrity and sperm concentration. Ejaculates were excluded from the statistical analysis if they exhibited any of the following characteristics: volume < 0.5 mL, mass motility, viability, or membrane integrity < 70%, sperm abnormalities > 10%, or sperm concentration < 1.5 × 10⁹ spermatozoa/mL. At the same time, some samples of semen were exposed to centrifugation at 4000 rpm for 10 min to obtain seminal plasma. Then, seminal plasma was well-maintained at -20 °C pending biochemical analysis. The levels of TAC, MDA, AST, and ALT in seminal plasma were also assessed using the previously established methods.

### Semen evaluation

Semen volume from each ram was documented utilizing a graduated collection tube in the same tube of collected semen. Mass motility was scored individually on a 0–100 scale based on wave motion readings from a microscope at low magnification (10X). Semen concentration was determined via a hemocytometer.

The sperm viability was assessed by the eosin/ nigrosine stain method according to Moskovtsev et al. [19] protocol. For preparing the eosin/ nigrosin solution, (5g of eosin and 0.9g of sodium chloride were dissolved in distilled water (100ml). The mixture was heated gently, followed by the addition of 10.0 g of nigrosine was added and kept under dark conditions until use). Samples (10 µL) from each group were incubated with the eosin/nigrosin solution (10 µL) for 2 min at 25°C and then smeared on hot glass slides for air-drying. At 400X magnification, at least 200 sperm cells were observed under a light microscope.

In the same scope, the number of sperm cells with abnormal tail morphology (coiled tail, broken tail, terminally coiled tail, double tail) and head morphology (microcephalic head, pear-shaped head, round short head, loose head, double head) was also recorded (as percentages), as previously described [19].

For assessing the membrane integrity (%) ratio, hypo-osmotic swelling (HOST) test as described by Khalil et al. [20] protocol was performed. In addition, this method was mentioned previously in detail in our previous works [21]. A total of 200 sperm cells were estimated for their swelling capability in HOST. Sperm cells with a swollen or coiled tail were considered to have an intact plasma membrane.

### Hormonal (ICSH, and testosterone) assessments

Testosterone and interstitial cell stimulating hormone were evaluated in both seminal plasma and blood serum. Plasma testosterone level was evaluated by ELISA using commercial ELISA kit provided from Abcam company (catalog number; ab215529; Cambridge, UK), following the protocol of Abbaticchio et al. [22]. Moreover, ICSH in the serum and seminal plasma were assessed using commercial kit (catalog number; ab222403; Cambridge, UK) according to Kjessler et al. [23].

### Total RNA isolation, cDNA synthesis, and quantitative real-time PCR (qRT-PCR)

Fresh semen ejaculates were collected from (*n* = 5 animals/group). After collection, all ejaculates were immediately kept in liquid nitrogen for total RNA isolation. Fresh semen ejaculates were thawed on ice and then centrifuged (500 × g for 10 min at 4 °C) to isolate the supernatant from the spermatozoa precipitation. Subsequently, the spermatozoa were washed, and somatic cells were lysed on ice for 20 min. Total RNA extraction from spermatozoa was carried out using the miRNeasy mini kit (Qiagen, Hilden, Germany) with the warm QIAzol lysis reagent method, following the protocol described by Ibrahim et al. [24].

A 20 μL reaction volume was prepared by mixing RNA template with 4 μL of Xpert cDNA Synthesis Supermix (5x) and 10 μL of RNase-free water. The thermocycler was programmed as follows: 37°C for 20 min, 60°C for 10 min, 95°C for 1 min, and then held at 4°C. The synthesized cDNA was validated using GAPDH primer in a PCR reaction and stored at -20°C. Gene-specific primers for *SOD1, GABPB1, CASP3, CAT, GPX1* and *GAPDH* were designed using Primer3 software (Table 2) [25].

**Table 2:** Gene’s names and details of primers used for qRT-PCR analysis

Gene	Sequence 5^\^ to 3^\^	Accession no	Product Size (bp)	Annealing ^o^C
*SOD1*	F: CAGTTTGGAGACAATACACAAG R: GGAATATTCTCCTGAGAGTGAG	NM_001145185.2	189	55
*CAT*	F: CATGACATTACCAGATACTCCA R: GATAAAGGACGGAAACAGTAGA	XM_004016396.5	231	56
*GPX1*	F: GCTCATTGAGAACGTAGCAT R: GTACTTCAGGCAATTCAGGAT	XM_004018462.5	175	54
*GABPB1*	F: GCAAGTATTAACAGTACCAGCA R: TAATTTCTGTCTGTAGGCCTCT	XM_042252469.1	232	57
*CASP3*	F: TGGACTCCGGAATATCCTTG R: AGGTTTCCCTGAGGTTTGCT	XM_015104559.3	153	55
*GAPDH*	F: ACAATACCCTCAAGATTGTCAG R: AGTAGAAGCAGGGATGATGTT	NM_001190390.1	212	55

Superoxide dismutase type 1 (SOD1), catalase (CAT), glutathione peroxidase 1 (GPX1), GA Binding Protein Transcription Factor Subunit Beta 1 (GABPB1), and caspase-3 (CASP3)

A 20 μL reaction volume containing Xpert Fast SYBR (uni) 2X Master mix, cDNA template, forward and reverse primers, and RNase-free water was used for the PCR reaction. The reaction was carried out using a StratageneMx 3000P instrument with the following program: 95°C for 10 min, 40 cycles at 95°C for 15 s, 60°C for 30 s, and 72°C for 30 s. Melting curve analysis was performed to verify amplification specificity. The stability of housekeeping genes (ACTB and GAPDH) across different groups was assessed using NormFinder software. Data analysis was performed using 2^−ΔΔCT^ method with standardization using a geometric mean of housekeeping genes [26].

### Statistical analysis

The data were checked for homogeneity of variance using Levene's test and for normality of distribution using the Shapiro–Wilk test. The data were analyzed using SPSS (Version 21.0; IBM Co., Chicago, USA) with a one-way ANOVA test. Pairwise comparisons between means were conducted using Tukey's test if a significant effect was detected. The results were reported as mean ± SEM. A significance level of P ≤ 0.05 was used to determine statistical significance. All percentages were transformed to arcsine values before the statistical analysis. Mean values were transformed into percentages and tabulated. Data on the expression of candidate genes were analyzed using one-way ANOVA followed by Dunnett’s multiple comparisons tests. The data were graphed using GraphPad 10.0.2 as mean ± SEM (GraphPad Software, Inc., San Diego, CA, USA).

## Results

### Characteristics of moringa oil nano emulsion

The mean particle size, zeta potential and polydispersity index (PDI) of moringa oil nano-emulsion are presented in (Table 3) and morphology of moringa oil nano-emulsion observed in Fig. 1 The mean particle size of moringa oil nano-emulsion was 120.4 nm and the zeta potential value was -18.3 mV.

**Table 3:** Particle size, Z potential and PDI of moringa oil nano-emulsion

Item	Particle size (nm)	Zeta potential (mV)	PDI
Moringa oil nano-emulsion	120.4	-18.3	0.537

### Physiological response

Table 4 shows the impact of NMO and MO on physiological responses such as RT, PR, and RR. There were no significant effects on RT, RR, and PR among all experimental groups (*P* > 0.05) during the research period in summer (June–August). The NMO group showed a tendency to reduce the pulse rate compared with other groups (*P* > 0.05) during the last two months of summer (July and August). Additionally, RR was decreased in the NMO group compared to the other groups, but this change was not statistically significant (*P* > 0.05). Overall, NMO slightly reduced the values of PR and RR without a statistically significant difference compared to other groups.

**Table 4:** Response of physiological attributes of rams as influenced by orally administered moringa oil (MO) or its nano-emulsion form (NMO)

Item	Experimental groups^1^			*P Value*
	CON	NMO	MO	
Rectal temperature (RT, °C)				
June	39.1 ± 0.09	38.8 ± 0.09	39.0 ± 0.03	0.255
July	38.8 ± 0.06	38.8 ± 0.03	39.1 ± 0.09	0.661
August	38.8 ± 0.17	38.8 ± 0.03	39.1 ± 0.03	0.422
Pulse rate (PR, beats/min)				
June	85.3 ± 0.88	88.0 ± 0.58	86.3 ± 1.33	0.129
July	86.3 ± 1.20	82.7 ± 0.88	85.3 ± 0.88	0.001
August	82.7 ± 1.20	80.3 ± 0.88	84.3 ± 0.33	0.385
Respiration rate (RR, breaths/min)				
June	30.0 ± 1.15	28.7 ± 0.88	29.7 ± 1.20	0.111
July	26.7 ± 1.20	27.3 ± 0.88	28.3 ± 0.88	0.209
August	27.0 ± 0.58	26.3 ± 0.67	27.3 ± 0.88	0.324

^1^CON; the first group was served as the control (without supplementation; orally administered with 1mL of distilled water). NMO: The 2^nd^ group was categorized as the NMO group, which given NMO at a dose of 1 mL/head/day; and MO; the 3^rd^ group was categorized as MO group, where rams received MO at a dose of 2 mL /head/day. All treated animals received the treatment orally, individually administered before the morning feeding. Data are presented as (Mean ± SEM)

### Impacts on growth indices

Table 5 clarifies the effects of MO and NMO on growth indices of Rahmani rams. There were no significant differences observed among all groups in terms of body weights (*P* > 0.05) at various time points during the experimental period (6–10 months of age). The average daily weight gain was slightly higher in the NMO group (0.164 kg/day) compared to the CON group (0.162 kg/day) and the MO group (0.158 kg/day), but this difference was not statistically significant (*P* > 0.005).

**Table 5:** Response of body weight and daily body gains of rams as influenced by orally administered moringa oil (MO) or its nano-emulsion form (NMO)

Item	Experimental groups^1^			*P* Value
	CON	NMO	MO	
Weight (kg)				
Initial weight (6 months of age)	31.9 ± 0.93	30.0 ± 0.83	29.8 ± 0.75	0.198
7 months of age	36.2 ± 1.11	34.1 ± 0.88	33.6 ± 0.89	0.166
8 months of age	41.8 ± 0.92	40.0 ± 0.92	39.3 ± 1.08	0.208
9 months of age	48.7 ± 1.10	47.0 ± 0.99	45.7 ± 1.22	0.240
10 months of age (Final weight)	56.2 ± 1.12	54.6 ± 0.86	53.5 ± 1.24	0.198
Weight gain (kg)				
6–7	4.3 ± 0.28	4.1 ± 0.25	3.8 ± 0.14	0.266
7–8	5.6 ± 0.24	5.9 ± 0.18	5.7 ± 0.26	0.647
8–9	6.9 ± 0.24	7. 0 ± 0.26	6.5 ± 0.24	0.301
9–10	7.5 ± 0.19	7.7 ± 0.22	7.8 ± 0.09	0.598
6–10 (total weight gain)	24.4 ± 0.21	24.6 ± 0.48	23.7 ± 0.52	0.317
Average daily weight gain (6–10)	0.162 ± 0.001	0.164 ± 0.003	0.158 ± 0.003	0.318

^1^CON; the first group was served as the control (without supplementation; orally administered with 1mL/head/day of distilled water). NMO: The 2^nd^ group was categorized as the NMO group, which given NMO at a dose of 1 mL/head/day; and MO; the 3^rd^ group was categorized as MO group, where rams received MO at a dose of 2 mL /head/day. All treated animals received the treatment orally, individually administered before the morning feeding. Data are presented as (Mean ± SEM, *n* = 5 replicates)

### Hematological profile

Table 6 exhibits the hematological changes in rams following oral administration of moringa oil or its nano-emulsion. The hemoglobin levels showed a tendency to increase in the MO group compared to the other treated groups (*P* > 0.05). There were no significant differences among treatments (*P* > 0.05) in terms of red blood cells, hematocrit, platelets, white blood cells, lymphocytes, and neutrophils values. The MO group exhibited the lowest MCV values compared to the other groups, while the CON group and NMO group showed similar results. In contrast, MCH was higher in all treated groups compared to the CON group (*P* = 0.003). Rams in the MO group had higher MCHC values than the CON group (*P* = 0.003). However, both the CON and NMO groups had similar results for MCHC.

**Table 6:** The hematology variables of Rahmani ram in response to orally administered moringa oil (MO) or nano-emulsion form (NMO)

Item^1^	Experimental group^2^			*P value*
	CON	NMO	MO	
Hb, g/dL	9.10 ± 0.10	9.98 ± 0.07	10.86 ± 0.67	0.05
RBCs, × 10^6^/mm^3^	5.24 ± 0.13	5.04 ± 0.08	5.47 ± 0.24	0.25
Hct, %	35.40 ± 0.56	33.97 ± 0.90	33.00 ± 0.79	0.16
MCV, μm3	67.66 ± 1.54^a^	67.37 ± 0.80^a^	60.40 ± 1.21^b^	0.009
MCH, pg	17.40 ± 0.31^b^	19.82 ± 0.24^a^	19.81 ± 0.40^a^	0.003
MCHC, g/dL	25.72 ± 0.18^b^	29.43 ± 0.71^ab^	32.86 ± 1.29^a^	0.003
PLT, × 10^3^/mm^3^	320.67 ± 6.69	289.00 ± 17.62	312.33 ± 22.67	0.44
WBCs, × 10^3^/mm^3^	8.37 ± 0.26	8.93 ± 0.72	7.70 ± 0.62	0.37
Lymphocytes, %	35.18 ± 0.58	38.80 ± 1.64	36.57 ± 1.77	0.27
Neutrophils, %	48.54 ± 0.58	50.22 ± 1.28	51.77 ± 2.02	0.34

^1^*Hb* hemoglobin, *RBCs* red blood cells, *Hct* hematocrit, *MCV* mean corpuscular volume, *MCH* (Mean corpuscular hemoglobin), *MCHC* mean corpuscular hemoglobin concentration, *PLT* platelets, *WBCs* white blood cells

^2^CON; the first group was served as the control (without supplementation; orally administered with 1mL/head/day of distilled water). NMO: The 2^nd^ group was categorized as the NMO group, which given NMO at a dose of 1 mL/head/day; and MO; the 3^rd^ group was categorized as MO group, where rams received MO at a dose of 2 mL /head/day. All treated animals received the treatment orally, individually administered before the morning feeding. Data are presented as (Mean ± SEM, *n* = 5 replicates)

### Blood biochemistry

The impacts of moringa oil and its nano-form on liver function, kidney function, lipid profile, and oxidative biomarkers are presented in Table 7. However, the values of ALT were not affected by the treatment, although a slight decrease in ALT levels was observed in both treated groups compared to the CON group (*P* = 0.2). Rams that received MO and its nano-form showed lower levels of AST (*P* = 0.001), and total cholesterol (*P* = 0.006) compared to rams in the CON group. Glucose and total proteins were higher in the MO group than in the CON and NMO groups (*P* < 0.01). There were no significant differences among treatments for albumin, bilirubin, creatinine, urea, total triglycerides, TAC, and MDA (*P* > 0.05). It is worth noting an increase in albumin and TAC levels in the MO and NMO groups, while the NMO group had lower urea and total triglycerides than the other groups (*P* > 0.05). Animals in the MO group had lower MDA levels and higher TAC levels than those in the NMO and CON treatments, Table 7.

**Table 7:** Changes in liver and kidney function, lipid profile and oxidative biomarkers of ram in response to orally administered moringa oil (MO) or its nano-emulsion form (NMO)

Item^1^	Experimental group^2^			*P* value
	CON	NMO	MO	
Liver Functions				
ALT, U/L	30.23 ± 1.70	28.03 ± 2.39	23.67 ± 2.67	0.20
AST, U/L	107.73 ± 4.16^a^	65.70 ± 5.14^b^	63.30 ± 4.49^b^	0.001
Albumin, g/dL	2.97 ± 0.18	3.59 ± 0.27	3.79 ± 0.04	0.05
Bilirubin, mg/dL	0.37 ± 0.03	0.33 ± 0.03	0.28 ± 0.02	0.20
Total protein, g/dL	5.56 ± 0.09^b^	6.26 ± 0.25^ab^	6.66 ± 0.11^a^	0.010
Kidney Functions				
Creatinine, mg/dL	0.97 ± 0.07	0.83 ± 0.03	0.77 ± 0.07	0.12
Urea, mg/dL	40.67 ± 2.40	35.67 ± 1.45	40.33 ± 0.67	0.14
Lipid profiles				
Total cholesterol, mg/dL	97.96 ± 3.28^a^	78.01 ± 2.89^b^	84.70 ± 1.91^b^	0.006
Total glycerides, mg/dL	58.49 ± 3.45	53.94 ± 1.00	57.56 ± 2.93	0.49
Glucose, mg/dL	52.80 ± 4.35^b^	52.00 ± 2.65^b^	71.60 ± 1.81^a^	0.007
Oxidative biomarkers				
TAC, ng/mL	0.82 ± 0.09	0.80 ± 0.07	0.93 ± 0.08	0.47
MDA, nmoL/mL	2.32 ± 0.50	2.46 ± 0.21	2.02 ± 0.17	0.65

^1^Alanine transaminase (ALT), Aspartate transferase (AST), total antioxidant capacity (TAC) and Malondialdehyde (MDA)

^2^CON; the first group was served as the control (without supplementation; orally administered with 1mL/head/day of distilled water). NMO: The 2^nd^ group was categorized as the NMO group, which given NMO at a dose of 1 mL/head/day; and MO; the 3^rd^ group was categorized as MO group, where rams received MO at a dose of 2 mL /head/day. All treated animals received the treatment orally, individually administered before the morning feeding. Data are presented as (Mean ± SEM, *n* = 5 replicates)

### Hormonal profile in serum and seminal plasma

Serum ICSH was significantly improved (*P* < 0.0001), while serum testosterone was significantly decreased (*P* < 0.0001) by NMO-treated group when compared with other groups (Table 8). Circulating blood testosterone and ICSH were similar in CON and MO groups. Ram given with MO had lower (*P* = 0.0008) values of seminal ICSH, while had greater levels of seminal Testosterone (*P* = 0.0007) when compared with NMO-treated and CON groups.

**Table 8:** Effect of orally administered moringa oil (MO) or its nano-emulsion form (NMO) on the concentration of ICSH and testosterone hormones in blood serum and seminal plasma

Item^1^	Experimental groups^2^			*P* value
	CON	NMO	MO	
ICSH, ng/dL				
Blood serum	0.12 ± 0.01^b^	0.28 ± 0.02^a^	0.12 ± 0.01^b^	< .0001
Seminal plasma	2.10 ± 0.17^a^	2.65 ± 0.20^a^	1.48 ± 0.09^b^	0.0008
Testosterone, ng/dL				
Blood serum	7.13 ± 0.99^a^	3.03 ± 0.28^b^	8.98 ± 0.42^a^	< .0001
Seminal plasma	0.30 ± 0.04^b^	0.23 ± 0.02^b^	0.89 ± 0.16^a^	0.0007

^1^interstitial-cell-stimulating hormone

^2^CON; the first group was served as the control (without supplementation; orally administered with 1mL/head/day of distilled water). NMO: The 2^nd^ group was categorized as the NMO group, which given NMO at a dose of 1 mL/head/day; and MO; the 3^rd^ group was categorized as MO group, where rams received MO at a dose of 2 mL /head/day. All treated animals received the treatment orally, individually administered before the morning feeding. Data are presented as (Mean ± SEM, *n* = 5 replicates)

### The activities of TAC, MDA, AST and ALT in seminal plasma

The impact of orally administering MO or NMO on the concentration of TAC, MDA, AST, and ALT in seminal plasma is outlined in Table 9. TAC levels displayed a slight increase in both the MO and NMO groups, but this rise was not statistically significant compared to the untreated group (*P* > 0.05). Conversely, the MO group showed lower levels of AST and MDA, whereas the ALT levels were the lowest in the NMO group (*P* > 0.05), as shown in Table 9.

**Table 9:** Effect of the orally administered moringa oil (MO) or its nano-emulsion form (NMO) on the concentration of TAC, MDA, AST, and ALT in seminal plasma

Item^1^	Experimental groups^2^			*P* value
	CON	NMO	MO	
TAC, mM/L	1.51 ± 0.08	1.59 ± 0.04	1.67 ± 0.02	0.13
MDA, nmoL/mL	2.53 ± 0.28	2.50 ± 0.13	2.10 ± 0.14	0.26
AST, U/L	106.40 ± 10.51	130.00 ± 20.00	87.80 ± 19.99	0.27
ALT, U/L	23.20 ± 5.57	19.00 ± 2.68	23.20 ± 3.97	0.73

^1^Alanine transaminase (ALT), Aspartate transferase (AST), total antioxidant capacity (TAC) and Malondialdehyde (MDA)

^2^CON; the first group was served as the control (without supplementation; orally administered with 1mL/head/day of distilled water). NMO: The 2^nd^ group was categorized as the NMO group, which given NMO at a dose of 1 mL/head/day; and MO; the 3^rd^ group was categorized as MO group, where rams received MO at a dose of 2 mL /head/day. All treated animals received the treatment orally, individually administered before the morning feeding. Data are presented as (Mean ± SEM, *n* = 5 replicates)

### Semen quality

The effects the orally administered moringa oil or nano-emulsion form on ram semen characteristics are presented in Table 10. Mass motility was significantly improved by dietary MO inclusion (*P* < 0.0001) compared with the NMO and CON groups. Moreover, viability and membrane integrity were significantly higher in the MO group compared with the other groups (*P* < 0.0001). Similar results for viability and membrane integrity were observed in both the CON and NMO groups (*P* < 0.0001). The sperm concentration was higher in the MO-supplemented group compared to the NMO group (*P* = 0.006), with no significant effect was detected compared with the CON group (*P* > 0.05).

**Table 10:** Effect of the orally administered moringa oil or nano-emulsion form on semen characteristics of ram

Item	Experimental groups^1^			*P* value
	CON	NMO	MO	
Volume, mL	1.1±0.10	1.1±0.06	1.3±0.07	0.130
Mass motility, %	80.0±1.17^b^	76.1±0.93^c^	83.6±0.97^a^	<.0001
Viability, %	80.1±1.15^b^	78.6±0.86^b^	85.1±0.84^a^	<.0001
Abnormality, %	3.9±0.29	3.9±0.27	3.3±0.29	0.220
Membrane integrity, %	79.5±0.75^b^	77.6±0.84^b^	83.1±0.98^a^	0.0002
Sperm concentration, 10^6^	235.5±11.26^ab^	213.9±7.71^b^	265.4±12.70^a^	0.006

^1^CON; the first group was served as the control (without supplementation; orally administrated with 1mL/head/day of distilled water). NMO: The 2^nd^ group was categorized as the NMO group, which given NMO at a dose of 1 mL/head/day; and MO; the 3^rd^ group was categorized as MO group, where rams received MO at a dose of 2 mL /head/day. All treated animals received the treatment orally, individually administered before the morning feeding. Data are presented as (Mean± SEM, *n* = 14 replicates)

### Antioxidant genes of ram semen

**Fig. 2:**
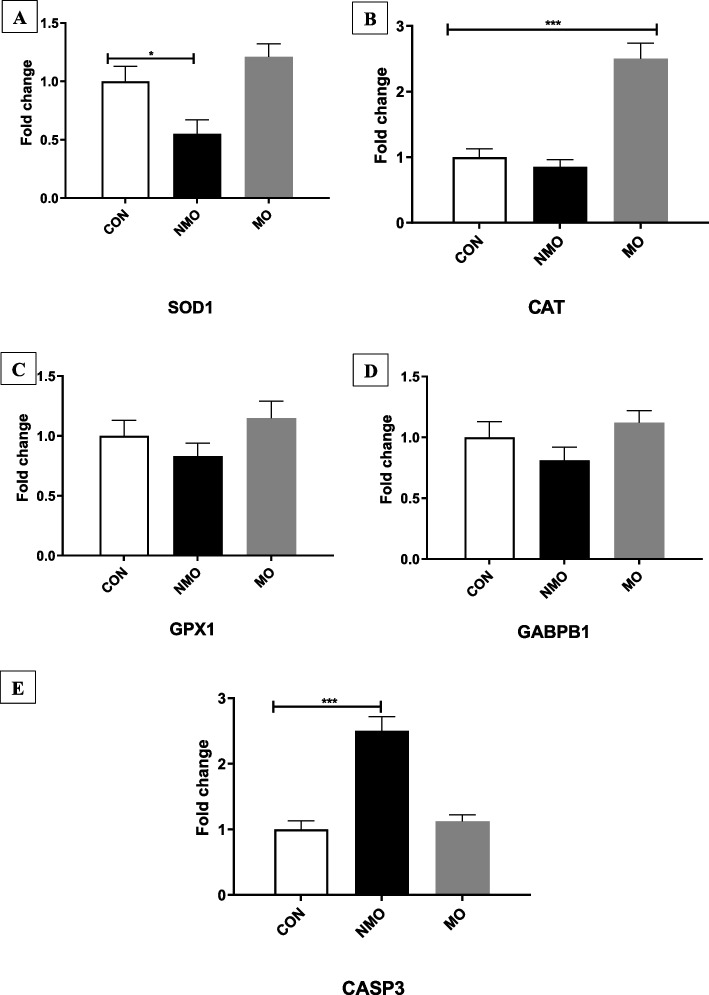
Relative abundance of some antioxidants [*SOD1* (Fig. 2A), *CAT* (Fig. 2B*), GPX1* (Fig. 2C), and *GABPB1* (Fig. 2D)] and apoptosis (*CASP3*, Fig. 2E) related genes in ram sperm cell. CON; the first group was served as the control (without supplementation; orally administrated with 1 mL/head/day of distilled water). NMO: The 2^nd^ group was categorized as the NMO group, which given NMO at a dose of 1 mL/head/day; and MO; the 3^rd^ group was categorized as MO group, where rams received MO at a dose of 2 mL /head/day. All treated animals received the treatment orally, individually administered before the morning feeding. Data are presented as (Mean ± SEM, *n* = 5 replicates)

The impact of MO or NMO on antioxidant genes of ram semen is illustrated in Fig. 2 (A-E). The NMO group exhibited a lower expression of *SOD1* (Fig. 2A) gene compared to the CON and MO groups. MO significantly upregulated the *CAT* (Fig. 2B) gene compared to the other groups (*P* < 0.001). The expressions of *GPx1* (Fig. 2C) and *GABPB1* (Fig. 2D) genes were similar in all groups (*P* > 0.05). The expression of *CASP3* (Fig. 2E) gene was highest in the NMO group compared to the other groups (*P* < 0.001).

## Discussion

Recently, essential oils have garnered attention as natural antioxidant agents due to their unique properties, including antimicrobial, antioxidant, anti-inflammatory, anti-apoptotic, anti-stress, appetite-stimulating, analgesic, and aphrodisiac effects [27]. In this study, the oral administration of MO or its nano-emulsion (NMO) significantly enhanced blood metabolites, semen characteristics, hormonal profiles, and total antioxidant capacity, while also reducing AST levels in ram serum. The data on growth indices and thermoregulatory response are similar among all groups. There is limited scientific research specifically investigating the effects of moringa oil on male fertility. Males can contribute to improving the reproductive capacity of a flock by providing good quality semen. Long-term exposure to various harmful effects, environmental issues [2, 28], and infectious elements [29] can worsen health, reduce body weight gains, increase oxidative stress, reduce semen quality, and subsequently reduce reproductive efficiency on farms [8, 30]. Supporting the immune system with food and herbal treatments can also improve gut microbiota, reduce inflammation, combat viral infections, and address nutritional imbalances [31]. Therefore, it is crucial to support the health, semen production, and welfare of rams [27]. Many reports have clarified that natural products derived from plants can boost animal health, the defense system, and reproductive capacity in males and may reduce sterility occurrence in the flock [2, 12]. Feeding suitable amounts of *Moringa oleifera* foliage (up to 25% of the diet) an enhance the body weight in growing goats under tropical conditions [32], while high levels of dietary *Moringa oleifera* foliage (< 25% of the diet) in goats diets may have detrimental effects on growth [32]. In our study, there was no statistical variation among all experimental groups, indicating no deleterious effects on growth.

Testosterone is a male hormone that plays a crucial role in male reproductive health. It affects the development of reproductive tissues, muscle mass, bone density, and libido. In this study, we observed that the MO administration improved the releasing of testosterone in both serum and seminal plasma. In rat, it was observed that *Moringa oliefera* leaf extract (MOLE) improved significantly the testosterone and reduced significantly the MDA after exposing to cadmium [33] or stress [34]. A low dose of extract enhanced sexual health in stress-exposed rats by lowering intromission latency and improving intromission frequency [34]. Moreover, increasing the numbers of interstitial cells of Leydig and spermatozoa were also found after MOLE treatments [34]. The extract's antioxidant properties could have led to the increase in Leydig cells and sperm count. In contrast, Ajuogu et al. [35] found that rabbit buck fed diets counting 5 and 10 g/kg of MOEL reduced significantly the serum testosterone.

Several studies have verified that *Moringa oliefera* can improve the sexual behavior via increasing the circulating testosterone hormone [36, 37]. This elevation induced by MO might attribute to its ability to improve the CYP3A4 enzymes, which is responsible for dietary compounds metabolism [38]. ICSH is a hormone primarily involved in the regulation of testosterone production in the testes. It's important to note that hormone regulation is a complex process influenced by various factors, and the specific effects of MO on hormone levels are not well-established. While in our research, the circulating ICSH was decreased by MO treatments indicating its utilization in the synthesis of testosterone, which has been confirmed by other scientists [39, 40]. The specific effects of MO on testosterone and other related sexual hormones levels have not been extensively studied, and the available research is limited.

Semen quality, which includes sperm count, motility, morphology, and overall reproductive health, can be affected by several factors such as dietary composition, environmental issues, and avoiding harmful substances. According to Syarifuddin et al. data, feeding bulls with *Moringa oleifera* meal (MOM) at 15% of the total dry matter intake significantly improved the libido, total motility, velocity, amplitude, and progressive motility of bull sperm [41]. In rabbits, semen volume, sperm count, and motility were significantly improved in a dose-dependent manner with increasing amounts of MOLE in the diet (0, 5, 10, and 15 g/kg) [35]. In rams, MOLE significantly enhanced semen volume, sperm counts, and seminal plasma antioxidants, including glutathione peroxidase, SOD, glutathione reductase, ALP, acid phosphatases, and TAC, when administered orally at a dose of 40 mg/kg daily [42]. Furthermore, studies have shown that freezing medium fortified with MOLE (0.5 mg/mL) significantly improved viability and progressive motility, while decreasing seminal plasma concentration of MDA and acrosomal defects and DNA fragmentation of sperm in cryopreserved ram semen [42, 43]. Recent research has demonstrated that oral supplementation of *Moringa oleifera* leaf ethanolic extract at 40 mg/kg BW significantly improved semen quality by increasing antioxidant capacity and altering the rumen microbiota and metabolites of cashmere goats [44]. This improvement may be attributed to MOLE significantly increasing the rumen levels of the steroid hormones testosterone and dehydroepiandrosterone, as well as the polyunsaturated fatty acids alpha-linolenic acid, gamma-linolenic acid, docosapentaenoic acid, and 9-S-hydroperoxylinoleic acid [44]. Another study showed that adding 10.0 mg/mL of *Moringa oleifera* seed extract (MOSE) to ram semen inhibited the growth of microorganisms, improved sperm characteristics, and enhanced the in vitro fertility of the semen [45]. MOLE significantly increased sperm concentration, motility, and viability, while altering the composition of the rumen microbiota by increasing the relative abundance of *Treponema* spp. and *Fibrobacter* spp. and reducing the relative abundance of *Prevotella spp*. Correlation analysis revealed that the genus *Prevotella* spp. was significantly negatively correlated with sperm concentration, motility, and viability [44].

Although there is limited research on MO specifically, most studies have been performed using MOLE in freezing medium or orally administered. More research is needed to determine whether MO can improve male fertility. MO is a vegetable oil extracted from the seeds of the moringa tree. It is a rich source of vitamins, minerals, and antioxidants [46]. It is well-recognized that MO is rich in nutrients and bioactive compounds [46], such as antioxidants and omega-9 fatty acids, which may have a direct impact on male fertility. MO has been proven to be effective as a hepatoprotective anti-oxidant, anti-inflammatory, anti-spasmodic, anti-diabetic, anti-microbial and cholesterol lowering agent [46]. *Moringa oleifera* may have positive effects on sperm quality and fertility-related parameters.

Blood glucose supports energy availability and is critical for spermatogenesis, influencing fertility rates and reproductive efficiency in animals. The administration of moringa oil significantly decreased blood glucose levels, as shown in the NMO group. These results are consistent with previous studies in rats [47–49] and rams [50]. This reduction in blood glucose may be due to the presence of phenolic compounds in *Moringa oleifera*. Some authors have suggested that this reduction is attributed to the oleic acids in *Moringa oleifera* [51]. Supplementation with approximately 20 g of MLE for at least 2 weeks could improve postprandial blood glucose in subjects with prediabetes or diabetes in human [52]. The capacity of the small intestine to uptake glucose is augmented due to an increase in the expression of *GLUT2* and *SGLT1*. However, the exact mechanism of how metformin reduces blood glucose is not fully understood.

Liver enzymes, such as AST, and ALT, are commonly measured in blood tests to evaluate liver health. High levels of these enzymes may indicate liver destruction or disease. Studies have shown that *Moringa oleifera* leaf extract (MOLE) has improved hepatic health in rats induced by a chemical toxin [33, 34, 39, 40]. Other reports have indicated that the extract helped normalize the levels of ALT and AST, suggesting potential liver-protective effects. Consistent with these findings [53, 54] demonstrated that MOLE effectively reduced elevated levels of ALT and AST, indicating a potential protective effect on the liver in goats [32]. While these studies provide some evidence of the hepatoprotective properties of MO, it is important to note that they primarily focused on MOLE rather than *Moringa oleifera* specifically. Additionally, more research, including well-designed animal studies, is needed to fully identify the consequences of moringa oil on liver enzymes and liver health.

Sperm cells, despite having built-in antioxidant defenses in seminal fluid, membranes, and cytoplasm, become vulnerable during dilution and cryopreservation due to partial loss and disruption of these protective systems [55]. Moringa oil is known for its rich antioxidant content [56]. Antioxidants are constituents that can assistance defend the body's cells from damage initiated by destructive particles identified free radicals [56]. Accumulation of free radicals can lead to oxidative stress, which is correlated with different health problems such as sterility, cancer, and aging. MO contains several antioxidants, including tocopherols (vitamin E), carotenoids (such as beta-carotene), and phenolic compounds. These antioxidants assistance counteract free radicals and decrease oxidative stress in the body. Research suggests that MO has antioxidant properties that may contribute to its potential health benefits [34, 42]. Similar to what was found in this study, several reports indicated that MOLE has potential effects in reducing the MDA and boosting the TAC in semen of several animal species [12, 42–45]. The antioxidant properties of MO may have various positive effects on overall sperm health and semen quality, including protecting against sperm damage, reducing lipid peroxidation, and supporting TAC.

Blood hematology is an indicator of animal health, helping to diagnose diseases and assess dietary interventions. In the present study, we found that there was no significant effect of orally administered MO or NMO on all blood hematological parameters except for MCV, MCH, and MCHC. MCV values were significantly decreased in rams given MO, while the NMO group had similar results compared with the control group. These results are partially agreed with Fathy et al. [50], who found that MOLE (5 g/100g diet) improved the MCV, and MCH in rams, but this improvement was not significant compared to the control group. This improvement in MCH and MCHC in MO or NMO revealed its ability to improve blood health and support to prevent anemia. Moringa supplementation has been shown to increase hemoglobin levels and improve other hematological parameters such as MCH, MCHC, and MCV values [57]. MCV is a measure used to determine the average size of red blood cells. These benefits are attributed to the iron content in moringa supplements. MCH and MCHC are an erythrocyte index that measures the average amount and concentration of hemoglobin in red blood cells, respectively. The MCHC and MCV values were increased in MO and NMO groups. Therefore, moringa can be considered a beneficial supplement for animals, particularly those at risk of developing anemia. These results are consistent with other previous studies in rats [58] and rams [57].

To confirm the antioxidative ability of MO or its nano-form in sperm cells, we have investigated the changes in *SOD1, GABPB1, GPX1,* and *CAT* as well as the apoptotic gene (*Caspase*-3) in sperm cells. Nano-emulsion of MO (NMO group) had lower expression of *SOD1*, while higher expression of *Caspase-3* compared to the CON and MO groups. MO significantly upregulated the CAT gene compared to the other groups (*P* < 0.001). After screening the literature, no previous studies have reported the use of MO or NMO to improve the reproductive health in animals. Recently, Hashim et al. [59] found that MO can mitigate carbendazim toxicity induced- reproductive failure in rats due to its antioxidative action. It has been indicate that the MO extracted from seeds has more beneficial effects than aqueous extract [60]. GPX1 is produced in the cauda and caput regions of the epididymis. It plays a role in regulating oxidative and reductive activities, influencing spermatogenesis and sperm maturation processes. The expression of caspase proteins plays a crucial role in apoptosis, serving as key effectors of cell death. Caspases are a group of cysteine proteases that are essential for the apoptotic process.

Moringa oil (MO) has antioxidant properties that can benefit reproductive health by protecting cells and regulating hormone production. It is rich in essential fatty acids necessary for successful reproduction. Research on the use of MO in animal reproduction suggests it may enhance fertility and improve outcomes. More studies are needed to understand how MO's antioxidants affect male fertility. While this study provides promising results, further research is necessary to solidify these findings. In vivo fertility trials could offer definitive confirmation of the beneficial effects of MO or NMO on sheep reproductive health. Additionally, exploring other metabolic markers and analyzing alterations in the rumen microbial community could provide deeper insights into the underlying mechanisms of action. Specifically, in vivo fertility trials using ram semen supplemented with MO could directly assess its impact on fertilization and embryonic development. Furthermore, investigating the effects of oral MO administration on the rumen microbiome could reveal potential interactions between MO and the gut microbiota that contribute to improved reproductive performance.

## Conclusion

Our findings suggest that moringa oil (MO, 2mL/day/head) and its nanoform (NMO, 1mL/day/head) did not notably impact growth indices or physiological responses in rams. However, they demonstrate potential benefits in enhancing blood health, semen quality, and supporting reproductive hormone levels compared to the control group. The precise mechanisms underlying the antioxidant effects of moringa oil in ruminants remain to be fully elucidated. Further research is needed to delve deeper into the specific components of *Moringa oleifera* oil that contribute to its antioxidant properties and their impact on sperm characteristics during cryopreservation. Future studies could also focus on identifying the active compounds and exploring their mode of action to optimize the utilization of moringa oil in reproductive health management.

## Data Availability

All data included in this study are presented in the form of tables and figures.

## Abbreviations

ALT: Alanine aminotransferase
AST: Aspartate aminotransferase
CAT: Catalase
GABPB1: GA-binding protein transcription factor subunit beta-1
GPx1: Glutathione peroxidase
Hb: Hemoglobin
ICSH: Interstitial-cell-stimulating hormone
MCH: Mean corpuscular hemoglobin
MCHC: Mean corpuscular hemoglobin concentration
MCV: Mean corpuscular volume
MDA: Malondialdehyde
MO: Moringa oil
NMO: Moringa oil nano-emulsion
OS: Oxidative stress
RBCs: Red blood cells
SOD1: Superoxide dismutase 1
TAC: Total antioxidant capacity
WBCs: White blood cells
